# Obituary Record

**Published:** 1878-07

**Authors:** 


### Obituary Record.

*C. A. Wunderlich.*—Sept. 25, 1877, æt. 62. Dr. Wunderlich was professor in the University of Leipzig, and best known by his work on "Temperature in Disease."

*M. Elie Gintrac.*—Dec., 1877, æt. 86. The most important of his works is a "Theoretical and Clinical Course on Pathology and Medical Therapeutics."

*Jean Bapt. Philip Barth.*—On the 3rd Dec., 1877, at Paris. æt. 72. His principal work was on auscultation and percussion, written in conjunction with M. Henri Pioger.

*Dr. Wm. Stokes*, the eminent Irish physician died in Dublin, Jan. 7, 1878, æt. 74.

*Ed. H. Clarke, M. D.*—In Boston, Nov. 30, 1877, æt. 57. Dr. Clarke was Professor of Materia Medica in Harvard College, and held an enviable position in Boston as consulting physician.

*Dr. James Blundell.*—At London, Jan. 15, 1878, æt. 87. Dr. Blundell was formerly Professor of Obstetrics at Guy's Hospital.

*Fleetwood Churchill, M. D.*—In Ireland, Jan. 31, 1878, æt. 70. Dr. Churchill was well known by his works, "Theory and Practice of Midwifery," "Diseases of Women, and "Diseases of Children."

